# Painful Todd’s: Post-ictal painful hemiparesis as an identifier of insular epilepsy

**DOI:** 10.1016/j.ebr.2025.100747

**Published:** 2025-02-02

**Authors:** Julian Larkin, Tudor Munteanu, Emma Dolan, Daniel J. Costello, Kieron Sweeney, Ronan Kilbride, Peter Widdess-Walsh

**Affiliations:** a Strategic Academic Recruitment Doctor of Medicine Programme RCSI University of Medicine and Health Sciences in Collaboration with Blackrock Clinic Dublin Ireland; b Department of Neurology and Clinical Neurophysiology, Beaumont Hospital, Dublin 9 Ireland; c Epilepsy Service, Cork University Hospital & College of Medicine and Health, University College Cork, Cork, Ireland; d Department of Neurosurgery, Beaumont Hospital, Dublin 9 Ireland

**Keywords:** Insular epilepsy, Todd’s paresis, Stereo electroencephalography, Cortical stimulation

## Abstract

The insula can generate seizures which mimic frontal, temporal and parietal epilepsies making electroclinical localization difficult. We report the case of a twenty-one-year-old woman who presented with seizure semiology of a left-sided painful somatosensory aura, progressing to bilateral tonic posturing and complex manual automatisms. She described a painful sensation and weakness affecting her left side following the offset of a seizure, with the pain consistenly outlasting the weakness. This would last from hours to days depending on the severity and duration of the seizure. Stereo-electroencephalography (SEEG) demonstrated seizure onset in the limen of the right insula. Extra-operative stimulation of the insula reproduced the clinical symptoms. She underwent radiofrequency thermocoagulation (RFTC) which has resulted in a significant reduction in seizure frequency. This case report describes a lateralized painful Todd’s phenomenon as a feature of insular epilepsy confirmed by SEEG and extra-operative stimulation.

## Highlights

• Careful attention to seizure semiology can help to distinguish insular epilepsy.
• Painful Todd’s paresis can localise seizure onset to the contralateral insula.
• Cortical stimulation confirmed the anatomical origin of this post-ictal phenomenon.

## 1 Introduction

The insula is a highly interconnected deep cortical structure with a role in multimodal somatosensory integration, emotional processing, and autonomic regulation [1], [2], [3].

SEEG-defined clinical features of insular seizures include retained awareness at onset, a sensation of laryngeal constriction or strangulation, contralateral paraesthesia which can be described as an uncomfortable or thermal sensation, and hypermotor behaviour [4], [5].

SEEG studies of insular seizures have demonstrated that clinical features differ according to the location of ictal onset in within the insula and the seizure propagation pattern [6], [7]. This is supported by functional mapping studies, which find that somatosensory symptoms typically arise from the posterior long gyri [8], [9]. Studies of structural connectivity show that regions of the insula are connected to their overlying cortical structures in a rostro-caudal pattern, which may influence seizure spread and clinical symptoms [10], [11].

Clinical suspicion for insular origin seizures is important in the pre-surgical planning of epilepsy through SEEG implantation [12], [13].

## 2 Methods

We performed a retrospective analysis of the case record including clinical information, EEG, imaging, and results of SEEG and extra-operative cortical stimulation. Relevant literature was reviewed through searches on PubMed. The patient provided informed consent, and this research was carried out in accordance with local ethics guidelines.

## 3 Case report

A twenty-one-year-old right-handed woman presented for evaluation of refractory focal epilepsy. Seizure onset was at nine years of age. She reported daytime diffuse left hemi-body somatosensory auras, described as painful. On occasion these would progress to complex motor activity, and rarely evolve to bilateral tonic-clonic seizures preceded by left head version. Seizures mostly occurred at night, with motor activity observed from sleep. Following a cluster of seizures, she would experience left hemi-body discomfort and paresis, with painful somatosensory symptoms continuing well beyond the resolution of motor weakness. Somatosensory symptoms could last from hours to days depending on the severity and duration of the seizure whereas motor weakness would typically resolve within one or two hours. She was experiencing focal seizures with impaired awareness a three times per week despite trials of eslicarbazepine, lacosamide, zonisamide, levetiracetam, lamotrigine, carbamazepine, and cenobamate. Her focal aware seizures.

Scalp video-EEG monitoring showed interictal broad right hemispheric sharp waves and seizures were of broad right hemispheric onset. MRI and PET of the brain were non-lesional. She underwent comprehensive neuropsychological evaluation which fell within the average range. Magnetoencephalography (MEG) and ictal single positron emission computed tomography (SPECT) were not available.

**Fig. 1:**
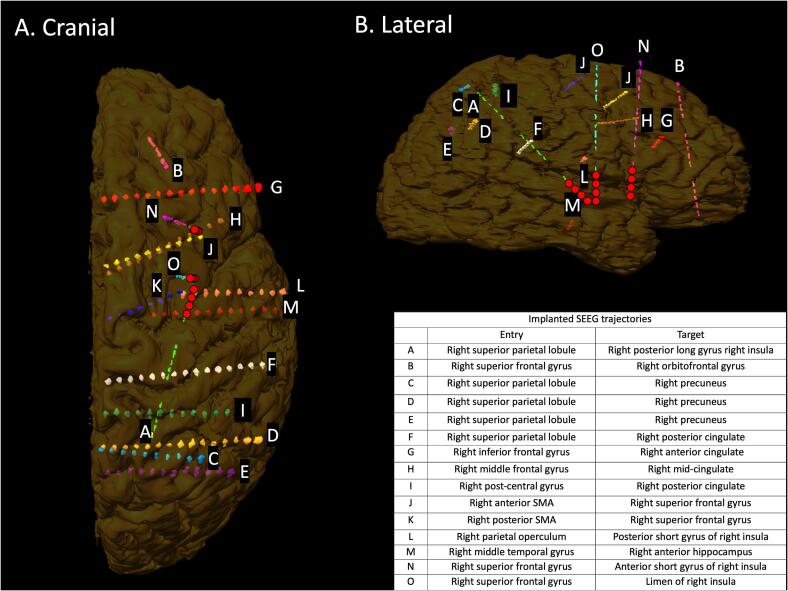
Volumetric MRI registration post-SEEG implantation. Volumetric T1-weighted reconstruction of depth electrodes (A through O) implantation trajectories in the frontal (A) and sagittal (B) planes. Electrode entry points and mesial targets are given in the inset table. Electrodes within the ictal onset zone are marked with red circles. (For interpretation of the references to colour in this figure legend, the reader is referred to the web version of this article.)

After discussion at an epilepsy surgery multidisciplinary meeting, she underwent SEEG with the hypotheses of an epileptogenic focus in the right supplementary motor, parietal or insular areas (areas known to cause sensory symptoms). Thirteen electrodes (A-M) (Dixi Medical, France) were implanted as shown in Fig. 1. In particular, the primary somatosensory cortex, supplementary somatosensory cortex, cingulate cortex, and insula were implanted due to the presence of somatosensory symptoms at onset. She underwent continuous video-EEG monitoring including cautious withdrawal of anti-seizure medications. Frequent interictal epileptiform abnormalities were recorded from the posterior long gyrus of the insula (A). Right anterior hippocampus (M) spikes were also recorded.

Eight typical seizures were recorded characterized by a vocalization at onset, progressing to bilateral tonic posturing and complex manual automatisms. This was followed by head version to the left, left hemiclonic movement, and progression to bilateral tonic-clonic seizure. She did not use the patient alarm to alert staff at the beginning of the seizures. After the seizure she described an uncomfortable, painful sensation affecting her left side, and there was focal weakness of her left upper limb on examination. Seizure onset was seen at the mesial contacts of the A electrode (posterior long gyrus of right insula). There was no involvement of the frontal motor regions. Two further electrodes (N and O) were implanted on day nine of monitoring to increase coverage of the anterior and mid-insula.

**Fig. 2:**
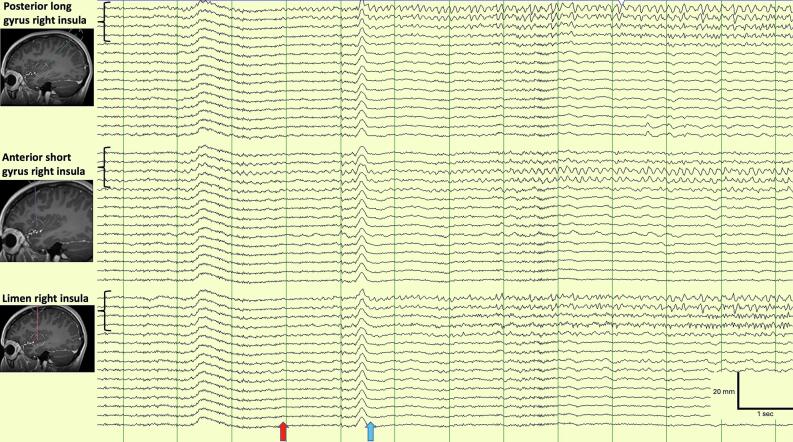
Example of seizure onset from the right insula. Seizure onset from the A, N, and O electrodes using a non-involved reference montage. There is paroxysmal fast activity arising from the limen and posterior long gyrus of the right insula at onset, with spread to the anterior short gyrus. Low frequency filter: 1 Hz, high frequency filter: 70 Hz, notch filter 50 Hz. EEG and clinical onset are denoted with the red and blue arrows respectively. (For interpretation of the references to colour in this figure legend, the reader is referred to the web version of this article.)

Interictal fast activity was recorded at electrodes N-1 to N-4 (right insula) and interictal spikes at N-1 to N-5, O-2 to O-5 (limen of right insula), and A-1 to A-4 (posterior long gyrus of right insula). For subsequent seizures, onset was at the limen of the insula (O-2 to O-5), with rapid spread to the posterior long and anterior short gyri of the right insula (A-1 to A-4). A representative example of seizure onset is shown in Fig. 2.

Extra-operative functional cortical stimulation of contacts was performed between pairs of contacts with a frequency of 1 Hz and pulse width of 0.3 ms. Stimulation within the limen (O) reproduced her typical post-ictal symptoms, a painful sensation and focal motor weakness on the left side at 10 mA. No after-discharges or EEG seizures occurred. There was a tingling of the left hand with no after-discharges with stimulation of the anterior short gyrus of the right insula (N) at 12 mA. Simulation at the posterior long gyrus of the insula (A) produced her typical sensory aura in her left arm at 10 mA, and EEG epileptiform discharges at 15 mA. Simulation at the posterior short gyrus of the insula (L) produced a sensation of heat in her neck at 10 mA, and a sensation of laryngeal constriction at 15 mA. There were no after-discharges.

**Fig. 3:**
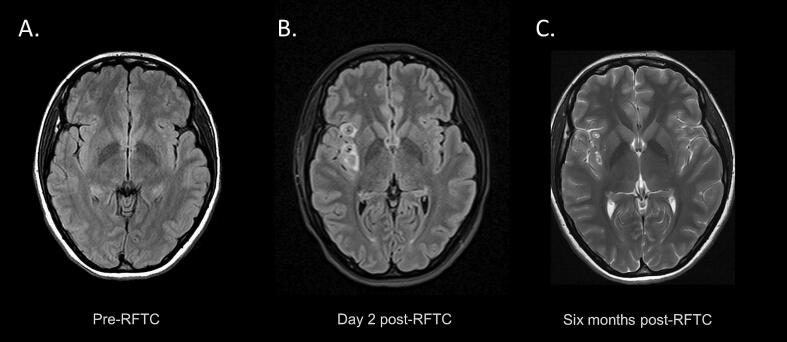
MRI changes following radiofrequency thermocoagulation. Axial T2/FLAIR-weighted MRI sequences showing insular structures before radiofrequency thermocoagulation (RFTC) (A), T2 signal hyperintensities surrounding ablation sites two days following RFTC (B), and residual changes six months after RFTC (C).

She underwent radiofrequency thermocoagulation (RFTC) of all insular grey matter contacts within the ictal onset zone. (Fig. 3). She had a period of seizure freedom of approximately one year after RFTC but subsequently experienced several breakthrough seizures in the context of intercurrent illness, likely due to partial disruption of the epileptic network within the insula. Two years after RFTC she reported a greater than seventy-five percent reduction in the frequency of her focal seizures with impaired awareness and a significant improvement in quality of life, with an Engel class IIA outcome. She has had no further bilateral tonic-clonic seizures and seizure semiology is unchanged. She was able to continue and graduate from university. She continues treatment with cenobamate, eslicarbazepine and lacosamide and does not wish to proceed with further surgical intervention at present. Insular resection is an option if seizures worsen in the future, but laser ablation is not available in our country.

## 4 Discussion

We describe the case of a patient who experienced a ‘painful Todd’s paresis’ following seizures with onset in the limen and posterior long gyrus of the insular cortex of the non-dominant hemisphere, which has not previously been reported. The presence of complex motor features and post-ictal left hemiparesis could support onset in the right hemisphere. However, careful attention to seizure semiology can provide clues to more accurate localization of the seizure onset zone. In this case, lateralized diffuse painful sensory symptoms supported onset in the contralateral insula, confirmed by SEEG recordings.

Extra-operative cortical stimulation of the insula reproduced the patient’s ictal somatosensory and painful symptoms with focal motor weakness. Previous studies of ictal recordings and electrocortical stimulation have indicated that painful somatosensory seizures are more often associated with seizure onset in the opercular-insular cortex than the primary somatosensory cortex [4], [5]. Lateralized painful hemiparesis as a postictal phenomenon has not previously been reported as an indicator of epilepsy originating from the contralateral insular cortex.

Typical Todd’s paresis results from seizures in frontal motor regions and is non-painful [12]. In this case, Todd’s paresis presented in association with a prolonged painful post-ictal somatosensory phenomenon, with seizure onset localized to the contralateral insula rather than the frontal lobe. Clinicians should elicit the presence of somatosensory painful symptoms in patients reporting Todd’s paresis, as it may be a useful identifier of insular epilepsy, that may be amenable to insular resection, RFTC, or laser interstitial therapy [13], [14].

## Ethical

The patient provided informed consent and this research was carried out in accordance with local ethics guidelines.

## CRediT authorship contribution statement

**Julian Larkin:** Writing – original draft, Visualization, Data curation. **Tudor Munteanu:** Writing – review & editing, Visualization, Data curation. **Emma Dolan:** Data curation. **Daniel J. Costello:** Writing – review & editing. **Kieron Sweeney:** Writing – review & editing. **Ronan Kilbride:** Writing – review & editing, Supervision, Conceptualization. **Peter Widdess-Walsh:** Writing – review & editing, Supervision, Conceptualization.

## Declaration of competing interest

The authors declare the following financial interests/personal relationships which may be considered as potential competing interests: PWW has been a paid speaker for Jazz Pharmaceuticals (epidiolex) and Angelini (ontozry). Remaining authors declare no conflicts of interest.
